# Activity of EGFR, mTOR and PI3K inhibitors in an isogenic breast cell line model

**DOI:** 10.1186/1756-0500-7-397

**Published:** 2014-06-25

**Authors:** Sharon Glaysher, Louise M Bolton, Penny Johnson, Christopher Torrance, Ian A Cree

**Affiliations:** 1 Translational Oncology Research Centre, Queen Alexandra Hospital, PO6 3LY Portsmouth, UK; 2 Horizon Discovery Ltd, 7100 Cambridge Research Park, Waterbeach, CB25 9TL Cambridge, UK; 3 Department of Pathology, University Hospitals Coventry and Warwickshire, Coventry, UK; 4 Yvonne Carter Professor of Pathology, Clinical Sciences Building, University Hospitals Coventry and Warwickshire, CV2 2DX Coventry, UK

**Keywords:** MTOR, Erlotinib, Gefinitib, ZSTK474, Sirolimus, MCF10, Breast cancer

## Abstract

**Background:**

The epidermal growth factor receptor family is expressed in breast cancer, and agents targeting this pathway have single agent effects (e.g. traztuzumab). Development of resistance may be due to the presence of alternative pathways, particularly activation of the PI3K/Akt/MTOR pathway. We have therefore examined the effect of inhibitors of this pathway (ZSTK474 and sirolimus) in combination with the epidermal growth factor (EGFR) inhibitors erlotinib and gefitinib in breast MCF10a isogenic cell lines with EGFR, BRAF, AKT, and PI3K mutations.

**Results:**

PI3K mutation conferred increased activity of EGFR inhibitors against MCF10a cells in comparison with the parental cell line and other mutations studied. Combination of EGFR inhibitors with either the PI3K inhibitor ZSTK474 or the MTOR inhibitor sirolimus showed increased activity.

**Conclusions:**

These results are encouraging for the use of combinations targeting the PI3K and EGFR pathway simultaneously.

## Background

**Figure 1:**
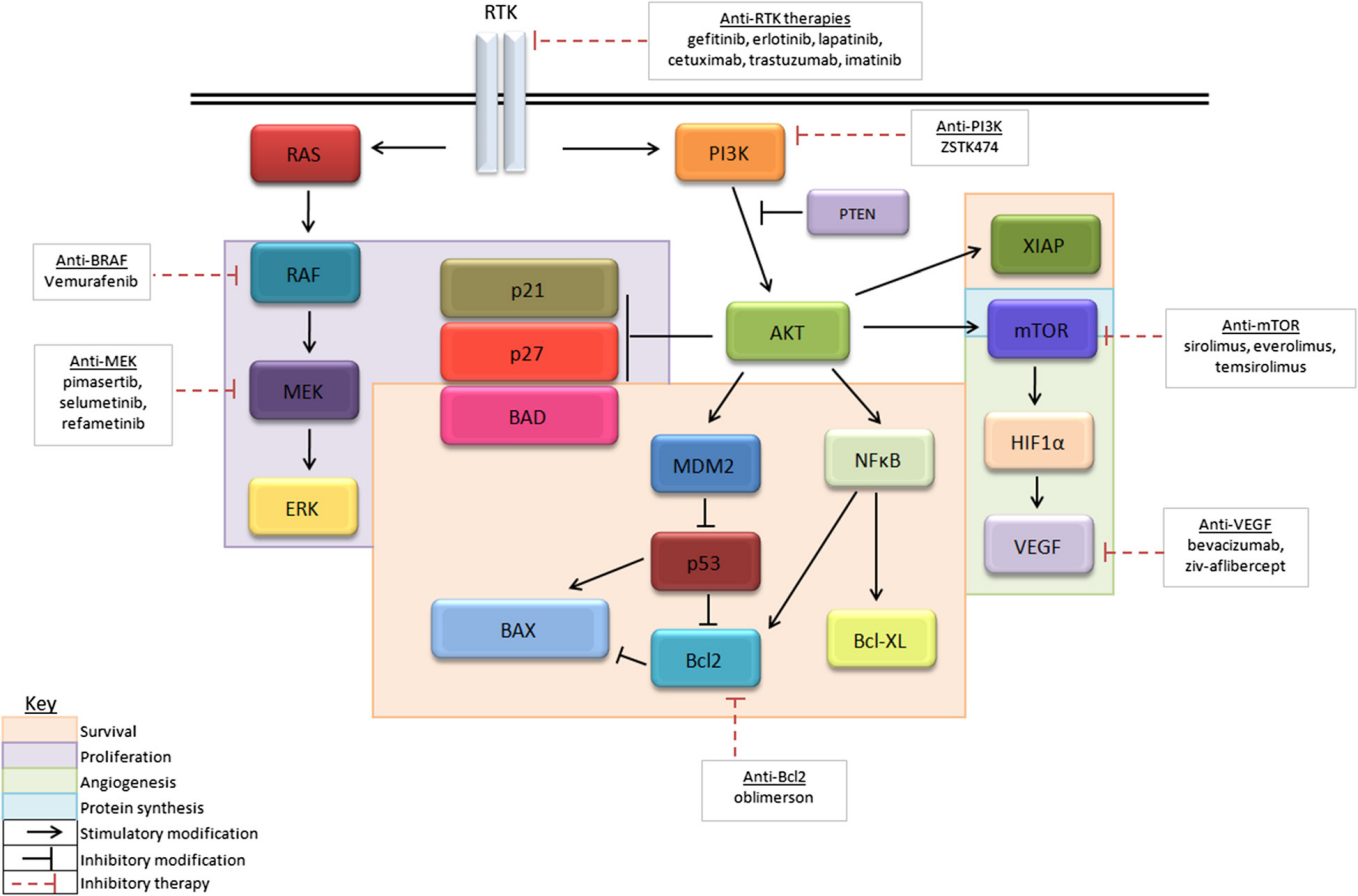
Human epidermal growth factor receptor and PI3K pathways in breast cancer.

Molecular alterations such as mutations or amplifications in Human Epidermal Growth Factor Receptors (HER) or downstream second messengers such as BRAF, AKT or PIK3CA can constitutively activate growth pathways in various cancers (Figure 1). Deregulated signalling through RAS-RAF-MAPK and PI3K-PTEN-AKT-mTOR pathways via such genetic alterations can result in unrestricted cellular proliferation and increased cell survival. It is for this reason that the development of drugs that specifically target these pathway components has become so popular.

Tumours exhibiting mutations within RTK pathway constituents (e.g. RAS, RAF, PIK3CA, PTEN, AKT, mTOR) or their upstream receptors have shown altered sensitivity to many different pathway targeted inhibitors including trastuzumab (Herceptin™) in breast cancer, gefitinib (Iressa™) in non small cell lung cancer (NSCLC), cetuximab (Erbitux™) in colorectal cancer (CRC) and vemurafenib (Zelboraf™) in skin melanoma when compared to their wild type counterparts. This has led to a stratified approach to cancer treatment within those cancers where a prognostic benefit has been identified with or without the presence of specific activating mutations.

Inhibition of a solitary signal transduction pathway is often inefficient due to the development of resistance through activation of alternative signalling cascades or receptor switching [1,2]. Data from NSCLC suggests that sensitivity to anti EGFR therapy is due to activating mutations within EGFR, which are not present in breast cancer. However, it has also been suggested that anti-EGFR strategies may also fail due to the presence of alternate activated pathways in which alternative activating mutations may be present. In breast cancer, HER2 amplification is used to guide the use of trastuzumab, an antibody to HER2, and lapatinib, a dual EGFR and HER2 small molecule targeted agent. Resistance to trastuzumab and lapatinib has been linked to aberrations of the PI3K pathway [3]. PI3K abnormalities including activating mutation of PIK3CA and loss of PTEN are common in breast cancer [3-6]. Other resistance mechanisms include activation of alternate pathways, particularly those involving IGF and HER3 [7-9].

To better understand how different activating mutations affect the sensitivity of RTK pathway targeted inhibition, we tested a set of human non-tumorigenic immortalized breast epithelial cells (MCF10a, obtained from Horizon Discovery Ltd) containing known activating mutations in EGFR, BRAF, AKT and PI3K along with the parental line which is wild type for these mutations against growth factor pathway inhibitors.

This isogenic cell line technology used here was initially developed by Di Nicolantonio and colleagues when a panel of isogenic human cell lines were created by employing homologous recombination (by knock-in) to characterize the response of the specific mTOR inhibitor everolimus to those cells containing specific mutations [10]. Using paired cell lines (isogenic and parental); drug sensitivity versus resistance was accurately assessed, with any phenotypic changes being a direct result of the introduced mutations. The DNA-modifications made to these commercially available cell lines are made within the endogenous gene so as to closely recapitulate the genetic events leading to the desired disease of study, or in our case to the effect of drugs targeting altered pathways.

In this study we have used a comparative approach. Mutant and wild type (Wt) MCF10a cells were tested for sensitivity against EGFR/PI3K/mTOR pathway inhibitors gefitinib (Iressa), erlotinib (Tarceva), sirolimus (Rapamycin) and ZSTK474, a pan-PI3K inhibitor as single agents and in combination [11].

## Methods

### Cell lines

Isogenic MCF10a cell lines with mutations in EGFR, BRAF, AKT and PI3K obtained from Horizon Diagnostics (Cambridge) were tested for sensitivity to these targeted inhibitors and compared with their parental MCF10a cell line. The MCF10a parent cell line (also available from ATCC) is immortalised, and was originally grown from adherent epithelia cells from a patient said to have fibrocystic disease. It was chosen by Horizon discovery for their X-man technology as it is easily transfected. It is non-tumorigenic, but does not exhibit senescence. Introduction of new mutations into this line does change its characteristics [12,13] and we believe it to be a good model of the generic effects of PI3K and EGFR inhibition in breast epithelium. Cell lines were grown in accordance to manufacturers’ guidelines. All were grown in DMEM:F12 medium with 5% horse serum, antibiotics (100 units/ml penicillin and 0.1 mg/ml streptomycin), 2 mM L-glutamine, 0.01 mg/ml insulin, 500 ng/ml hydrocortisone, 100 ng/ml cholera toxin, and 20 ng/ml EGF. Cell lines were grown adherent to plastic in 75 cm^2^ tissue culture flasks (Fisher Scientific UK; TKV-123-031 L and passaged at regular intervals when they reached 90% confluence. Cells for chemosensitivity assay were harvested by trypsinisation and washed twice in serum-free complete assay medium (CAM) before plating for chemosensitivity assays.

### ATP-TCA

The ATP-Based tumour chemosensitivity assay (ATP-TCA) was performed as previously described [14,15].

(a) Preparation of chemotherapeutic agents

Gefitinib, erlotinib, ZSTK474 and sirolimus were purchased from LC Laboratories (Massachusetts, US). All drugs were diluted in CAM (Innovative Diagnostik-systeme) to concentrations thought to be clinically achievable (gefitinib 0.06-2 μM, erlotinib 0.2-6.5 μM, ZSTK474 0.07-2.2 μM, sirolimus 0.06-2 μM). Combinations were tested by simultaneous addition. All of the chemotherapeutic drugs or combinations were tested in triplicate at 6 dilutions in 96-well round-bottomed polypropylene microplate (Corning Life Sciences, UK; 3790), allowing four drugs or drug combinations were tested. Two controls were included in one row of each plate: a no drug control consisting of media only (MO) and a maximum inhibitor (MI) control which kills all cells present giving a zero ATP count.

(b) ATP extraction and measurement

ATP was extracted from cells by the addition of 50 μl of ATP extraction reagent (DCS) to each well of the 96-well plate. Plates were incubated at room temperature for a minimum of 20 minutes and a maximum of one hour before the ATP was read. The ATP in the wells was measured using a luciferin-luciferase counting reagent as previously described [14,15]. Light output was measured using a Berthold Diagnostic Systems MPL1 luminometer (Berthold Diagnostic Systems, Pforzheim, Germany). All luminescence measurements were performed using the manufacturer’s instructions and an ATP standard curve run before each read using Adenosine 5’-triphosphate standard disodium salt hydrate (Sigma).

(c) Viability analysis

The data produced from each ATP-TCA plate was entered into an Excel (Microsoft®) spreadsheet that calculated the percentage tumour growth inhibition at each concentration, the IC50, and IC90 (concentration of drug required to cause 50% and 90% inhibition) for each drug as described in [14,15]. The percentage tumour growth inhibition at each drug concentration was used to plot curves for each drug or combination. As the variation between the wells that were averaged to calculate the percentage tumour inhibition is small (typical coefficient of variance of less than 10%), error bars have not been included on most graphs as they are usually smaller than the markers on the graphs. The percentage tumour growth inhibition was calculated as follows:

$$\begin{array}{ll} \%\;\mathrm{Inhibition}\;=\;1.0\;- & \;\left(\mathrm{Test}-\mathrm{MI}\right)\;\times 100\\ & \left(\mathrm{MO}-\mathrm{MI}\right) \end{array}$$

Test = mean counts for test drug wells

MI = mean counts for maximum inhibitor wells

MO = mean counts for medium only wells

To allow comparison between different cell lines, a sensitivity index (Index_SUM_) was used to calculate sensitivity based on percentage inhibition for each drug or combination, where IndexSUM = Sum(Inhibition200….6.25%).

The effects of drug combinations compared with their single agent counterparts were analysed using combination indices. Combination indices (CI) calculated by the Chou and Talalay [16] methods were determined at 50% and 90% cell death. These were defined as follows:

$$\begin{array}{l} \mathrm{CIA}+B\;=\;\left[\left(\mathrm{DA}/A+B\right)/\mathrm{DA}\right]\;+\;\left[\left(\mathrm{DB}/A+B\right)/\mathrm{DB}\right]\;+\\ \;\left[\alpha \left(\mathrm{DA}/A+B\;x\;\mathrm{DB}/A+B\right)/\mathrm{DADB}\right] \end{array}$$

Where CIA + B = CI for a fixed effect (F = 50% or 90%) for the combination of cytotoxic A and cytotoxic B; DA/A + B = concentration of cytotoxic A in the combination A + B giving an effect F; DB/A + B = concentration of cytotoxic B in the combination A + B giving an effect F; DA = concentration of cytotoxic A alone giving an effect F; DB = concentration of cytotoxic B alone giving an effect F. α = parameter with value 0 when A and B are mutually exclusive and 1 when A and B are mutually non-exclusive. The combination index CI calculates synergism <0.8; additivity >0.8 and <1.2; antagonism >1.2 [17].

### Mutation analysis

For DNA extraction, the Ambion® RecoverALL™ Nucleic acid Isolation kit using their total nucleic acid isolation protocol was used with RNase, according to the manufacturer’s instructions. Samples were quantified and purity checked using 1.5 μl of each undiluted sample with the NanoDrop spectrophotometer.

Rapid mutation screening for common mutations in EGFR, PI3K and BRAF was performed using Therascreen (Qiagen) kits, according to the manufacturer’s instructions. PCR was performed in an AB 7500 Fast Dx PCR machine and results downloaded to an Excel spreadsheet for analysis.

## R**esults**

The ATP-TCA was used to determine the effect of EGFR inhibitors (gefitinib and erlotinib) alone and in combination with inhibitors of the Akt/PI3K/mTOR pathway.

### Effect of single agents on isogenic MCF10a cell lines

(a) EGFR inhibitors

**Figure 2:**
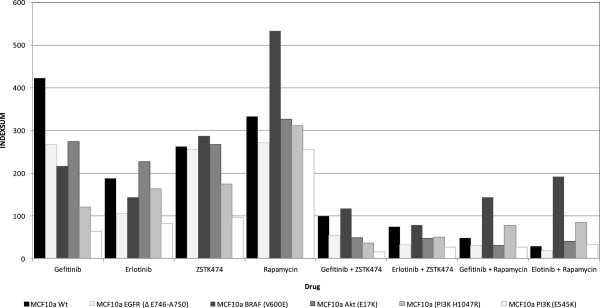
**Sensitivity of gefitinib, erlotinib, ZSTK474 and sirolimus alone and in combination on the parental MCF10a breast cancer cell line compared with isogenic clones with EGFR, KRAS and PI3K mutations.** A sensitivity index of zero = complete inhibition, 600 = no inhibition.

The parental MCF10a cell line showed greater resistance to gefitinib than those with mutations in EGFR, KRAS, PI3K, BRAF and AKT (Figure 2). Greatest sensitivity was seen within the PI3K mutated cells where Index_SUM_ values decreased from a relatively resistant index of 423 to 120 and 64 for the H1407R and E545K mutations of PI3KCA respectively. MCF10a cells were more sensitive to erlotinib (Index_SUM_ = 188) than gefitinib (Index_SUM_ = 423). Changes in sensitivity caused by the mutations were less pronounced in response to erlotinib exposure. The PIK3CA mutation E545K still became the most sensitive phenotype, but the effect of an AKT mutation produced a slightly more resistant phenotype (Index_SUM_ = 227) when compared with the parental line (Index_SUM_ = 188). However, both showed sufficient cellular inhibition to be classed as active agents in this setting with an index_SUM_ <300 representing 50% inhibition across the range of concentrations tested [18].

(b) PI3K inhibitor

All mutated MCF10a cell lines showed sensitivity to the PI3K inhibitor ZSTK474 (Index_SUM_ < 300) with EGFR and AKT mutations having no effect on the activity of this agent. Results with these mutations were similar to the parental line. PI3K mutations conferred greater sensitivity to ZSTK474 compared with the parental line, while cells containing the BRAF mutation V600E showed a more resistant phenotype.

(c) mTOR inhibitor

Sirolimus was the least active of the four inhibitors tested (Figure 2). Only MCF10a cells harbouring EGFR and PIKCA (E545K) mutations showed sensitivity with Index_SUM_ of 272 and 254 respectively which was an increase compared with the parental line Index_SUM_ of 333. BRAF mutation containing cells (V600E) again showed the most resistant phenotype with an Index_SUM_ of 533.

### Effect of combinations on isogenic MCF10a cell lines

Combination of the EGFR inhibitors with either ZSTK474 or sirolimus resulted in greatly increased cellular inhibition (Figure 2), with sensitivity Index_SUM_ values in all MCF10a cell lines below 200 and corresponding CI indices (Table 1). The effects of mutations on sensitivity were observable with cells containing BRAF mutations, though these showed less sensitivity to combinations when compared with the parental line. MCF10a cells containing the PI3K mutation H1047R showed little sensitivity to single agent sirolimus, and in combination with EGFR inhibitors were still seen to be more resistant than their parental counterpart, and were more resistant than MCF10a cells containing the PIK3CA E545K mutation.

**Table 1:** Chou and Talalay combination indices for each cell line examined

	**Gefitinib + ZSTK474**	**Erlotinib + ZSTK474**	**Gefitinib + Rapamycin**	**Erlotinib + Rapamycin**	**Gefitinib + ZSTK474**	**Erlotinib + ZSTK474**	**Gefitinib + Rapamycin**	**Erlotinib + Rapamycin**
**Cell Line**	**C150**				**C190**			
MCF10a Wt	0.23	0.58	0.02	0.28	0.47	0.42	0.03	0.07
MCF10a PI3K (H1047R)	**1.15**	**0.93**	**1.09**	**0.89**	0.18	0.39	0.46	0.40
MCF10a PI3K (E545K)	**1.15**	**1.41**	**1.52**	**1.30**	0.41	0.75	0.32	0.44
MCF10a EGFR (Δ E746-A750)	0.40	**0.93**	0.60	**1.10**	0.30	0.27	0.04	0.09
MCF10a BRAF (V 600E)	**0.93**	**1.18**	0.59	**1.07**	0.35	0.19	**1.04**	**1.33**
MCF10a Akt (E17K)	0.37	0.51	0.20	0.33	0.18	0.18	0.05	0.08

The combination index CI calculates synergism <0.8; additivity >0.8 and <1.2; antagonism >1.2. Synergism is noted in the wild type cell line, and in the Akt mutated line while there is some indication of antagonism in one PI3K mutated line.

### Mutation status of MCF10a cell lines

MCF10a cell lines included mutations in EGFR (exon 19 deletion E746-A750), BRAF (c.1799 T > A–V600E), PI3K (c.3140A > G–H1047R and c.1633G > A–E545K) and AKT (c.49G > A–E17K). All cell line mutations were confirmed where Therascreen ARMS kits for the relevant mutation were available (Qiagen Ltd, Manchester): confirmation was obtained for all but the AKT mutation, which was not available as a Therascreen kit. The parental MCF10a cell line was also screened against all available mutation tests to confirm mutation exclusion.

## Discussion

Variation in sensitivity was seen with different mutations for all four single agents. Cells with PIK3CA mutations H1047R and E545K showed greatest single agent sensitivity to the PI3K inhibitor. Interestingly, cells with these mutations also showed greater sensitivity to gefitinib and erlotinib than cells with the EGFR mutation (exon 19 deletion), which are associated with sensitivity to EGFR inhibitors in NSCLC. Mutations in PI3K (H1047R) have been shown to enhance HER2-mediated transformation by amplifying the ligand-induced signaling output of the HER family of RTKs [19]. It could be therefore be assumed that because PIK3CA mutations drive HER related receptor addiction in these cells it would make them more susceptible to RTK inhibition (as seen here with gefitinib and erlotinib) (Figure 1).

Cells containing the PIK3CA mutation E545K were found to be more sensitive than those with the PIK3CA mutation H1047R, suggesting that not all activating mutations found within a given gene will result in the same activity to targeted therapy. Equally cells with activating mutations can react differently to inhibitors of the same target. This is seen here with EGFR mutants as cells with EGFR mutation were significantly more susceptible to erlotinib compared with gefitinib. In reality not all activating mutations within a given gene are comparable in their effect, as has been shown in other tumour types. For instance, it has been suggested that not all KRAS mutations in colorectal cancer are equally effective in conferring resistance to anti-EGFR antibodies [10].

Least inhibition from exposure to the mTOR inhibitor sirolimus was seen in the presence of the BRAF mutation V600E. This is unsurprising given that inhibition of mTOR by sirolimus drives signalling through alternative pathways (AKT/PI3K or MEK/ERK) resulting in hyperactivation of the RAS/RAF/MAPK pathway due to the presence of the BRAF mutation [20-22].

The most effective combinations were of EGFR and PI3K inhibitors. In the presence of gefitinib, PI3K signalling can be maintained by an activated IGF1R pathway [23,24]. Therefore it could be suggested that for single agent anti-EGFR therapy to remain effective it would require cells to have EGFR dominant HER signalling in the absence of IGFR signalling pathways. In anti-EGFR resistant tumours showing this profile further alternate signalling mechanisms may be employed, including continued signalling via Met by driving HER3 dependent activation of PI3K. Therefore the combinational strategy of EGFR and PI3K takes advantage of any further downstream signalling through PI3K and is found to be effective despite which mutation is present.

## Conclusions

In breast cancer, there has been considerable interest in the combination of PI3K pathway inhibitors with anti-HER2 therapy. Data from different cell lines have been used to justify this [25], but this is to our knowledge the first report using a single parental cell line. Clinical trials are now in progress, and early results are encouraging for the combination of trastuzumab and mTOR inhibitors [26]. PI3K inhibitors are also entering clinical trials in combination with HER2 inhibitors [27]. The data presented here support these trials, and support the importance of full molecular characterisation of tumours where possible, suggesting that a more effective strategy in the use of pathway-targeted treatment may come from using the agents in combination to secure a strong signalling blockade.

## Competing interests

CT was a Director of Horizon Discovery Ltd when this work was performed, and IC is a Director of CanTech Ltd. Neither author will gain financially from this publication. The other authors declare that they have no competing interests.

## Authors’ contributions

CT and IC conceived and designed the study. SG, LM and PJ carried out the cell culture and molecular studies. SG and IC performed the statistical analysis and wrote the initial draft. All authors participated in review of drafts and approved the final manuscript.
